# Non-Intrusive Monitoring of Vital Signs in the Lower Limbs Using Optical Sensors

**DOI:** 10.3390/s25020305

**Published:** 2025-01-07

**Authors:** Joana Simões, Regina Oliveira, Florinda M. Costa, António Teixeira, Cátia Leitão, Pedro Correia, Ana Luísa M. Silva

**Affiliations:** 1 Institute for Nanostructures, Nanomodelling and Nanofabrication (i3N), Department of Physics, University of Aveiro, 3810-193 Aveiro, Portugal; joanasimoes02@ua.pt (J.S.); regina.oliveira@ua.pt (R.O.); flor@ua.pt (F.M.C.); catia.leitao@ua.pt (C.L.); pmcorreia@ua.pt (P.C.); 2 Institute of Electronics and Informatics Engineering of Aveiro (IEETA), Department of Electronics Telecommunications & Informatics, University of Aveiro, 3810-193 Aveiro, Portugal; ajst@ua.pt

**Keywords:** invisible health monitoring, heart rate, respiratory rate, optical sensor, photoplethysmography, lower limbs

## Abstract

Invisible health monitoring is currently a topic of global interest within the scientific community. Sensorization of everyday objects can provide valuable health information without requiring any changes in people’s routines. In this work, a feasibility study of photoplethysmography (PPG) acquisition in the lower limbs for continuous and real-time monitoring of the vital signs, including heart rate (HR) and respiratory rate (RR), is presented. The proposed system uses two MAX30102 sensors to obtain PPG signals from the back of the thigh. As proof of concept, tests were conducted in 17 volunteers (age group between 22 and 40 years old, twelve females and five males), and the results were compared to those of reference sensors. A Pearson correlation coefficient of r = 0.92 and r = 0.77 and a mean difference of 1.2 bpm and 0.9 rpm for HR and RR, respectively, were obtained between the developed system and reference. System accuracies of 95.9% for HR and 91.3% for RR were achieved, showing the system viability for vital sign monitoring of the lower limbs.

## 1. Introduction

Monitoring health at home typically relies on medical devices and wearables that provide accurate measurements of various health parameters. However, they are not always ideal or practical for everyone, especially older adults, who may encounter difficulties in adopting or using these technologies. On the other hand, many of these devices require human intervention, imposing changes in users’ routines, which limits adherence and increases the probability that users will gradually stop taking measurements. Furthermore, incorrect and inconsistent use of these systems can lead to long gaps in data collection, affecting the effectiveness of health monitoring [1,2].

Currently, passive and invisible vital sign monitoring technologies are being developed to be imperceptibly integrated into people’s daily lives, allowing for unobtrusive and continuous health monitoring in the home environment [3,4]. Almost all everyday objects around us have the potential to be converted into intelligent systems by integrating sensors and algorithms with automatic data evaluation [5]. Furthermore, their unobtrusive design may promote greater societal acceptance [6,7]. This passive and longitudinal monitoring has the potential to significantly revolutionize the healthcare system by enabling new proactive and preventive approaches [1,2].

Given that modern society spends a significant portion of time sitting—whether working from home, studying, engaging in leisure activities, eating, or even using the bathroom—the lower limbs, specifically the posterior region of the thigh, can be an optimal choice for continuous monitoring of vital signs. This allows us to extend measurements of health parameters to seated positions and to embed invisible health monitoring systems into less explored everyday objects such as chairs, sofas and toilets. On the other hand, it is important to note that the literature on monitoring devices for the lower limbs is scarce, with most studies focusing on devices designed for the upper limbs (e.g., fingers), ears, and chest [8,9].

Attempts have been made to monitor vital signs from the lower limbs by using systems to measure the electrocardiogram (ECG), balistocardiogram (BCG) and/or photoplethysmogram (PPG) signals. Among these techniques, PPG stands out due to the possibility of simultaneously measuring three vital signs, namely heart rate (HR), respiratory rate (RR) and oxygen saturation. Despite its versatility and widespread application in other regions of the body, the use of PPG to monitor vital signs from the lower limbs remains barely explored in the literature.

An example of this use is the work of Erts et al. [10], which explored the use of PPG signals acquired in fingers and toes for the early detection of arterial stenosis and other vascular conditions, and the work of Allen et al. [11], who uses the PPG signal obtained from the ears, hand and toes to check for peripheral vascular disease. Another significant contribution to this topic was made by Kim et al. In 2004, the group implemented a multichannel PPG system to study the optimal sites to obtain a physiological signal from the thigh. Twelve positions were studied, and tests were conducted with volunteers to draw conclusions. Although this study provides interesting preliminary findings, the authors performed a rudimentary comparison by manually counting the peaks of the PPG and ECG signal, which compromises the reliability of the study’s conclusions [12]. In 2006, the same researchers developed a system capable of ECG and PPG measurements, allowing the collection of BP data from the user. The ECG was obtained through the contact between three copper-coated electrodes and the user’s thigh, while the PPG signal was obtained through a reflective optical sensor [13].

This paper introduces a novel approach based on PPG, designed for real-time and longitudinal measurement of vital signs in the lower limbs, namely HR and RR. This research aims to explore and expand the understanding of PPG signal acquisition in less-studied body sites and, therefore, contribute to the literature with relevant findings to guide future research.

## 2. Photoplethysmography

**Figure 1:**
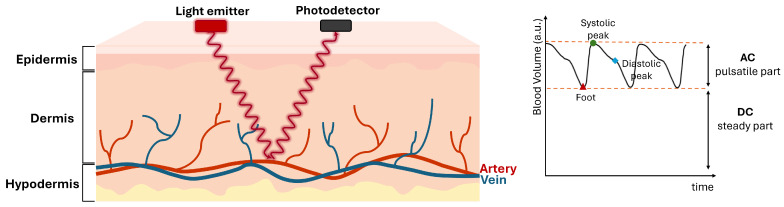
PPG principle: The light emitted by the optical sensor (light emitter) penetrates the skin and blood vessels. The reflected light reaches the photodetector. The PPG signal waveform consists of the inverted reading of the sensor to express the blood volume instead of the intensity of the light measured.

One of the most common health monitoring methods is the PPG (Figure 1), which consists of a non-invasive optical technique used to detect blood volume changes during the cardiac cycle. A PPG sensor consists of a light-emitting diode (LED) as the light source (visible and/or infrared (IR)) and a photodetector (PD) capable of detecting the transmitted or reflected light by the blood in the vessels [14,15,16]. The amount of light received by the PD depends on the blood volume in the light path. During the systole, the blood volume increases, leading to a higher light absorption, and consequently less light reaching the PD. Conversely, during the diastole, the blood volume decreases, resulting in less light absorption and more light reaching the PD [17]. Thus, this type of sensor can monitor the blood volume changes by detecting fluctuations in light absorption.

The PPG signal consists of two main components: the alternating component (AC), corresponding to the pulsatile part, and the direct component (DC), which is influenced by factors such as the steady volume of blood, skin thickness, sensor–skin distance, and is responsible for the offset of the signal [16].

## 3. Materials and Methods

### 3.1. Hardware Setup

**Figure 2:**
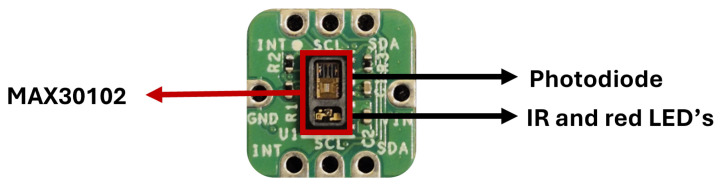
MAXREFDES117 module equipped with a MAX30102 optical sensor.

The MAXREFDES117 from the Analog Devices (Wilmington, MA, USA) [18] module was used, which incorporates the MAX30102 optical sensor [19], shown in Figure 2. It comprises two internal LEDs: one red (660 nm) and one IR (880 nm), a photodiode, and low-noise electronics with ambient light rejection, which helps maintain measurement accuracy across different lighting conditions. It presents other ideal features. It presents ideal features for this application, such as small size, high signal-to-noise ratio (SNR), low power consumption, and is both cost-effective and suitable for mass production [19].

**Figure 3:**
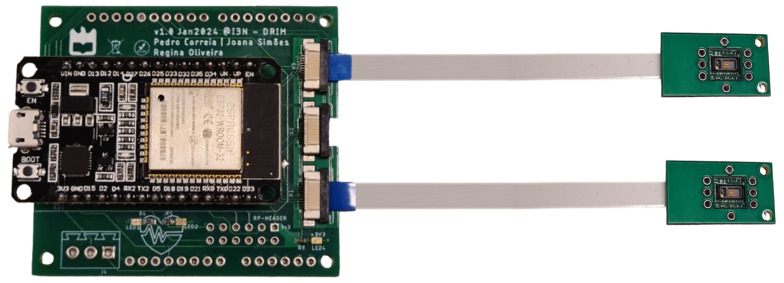
Hardware setup used including the two front-end sensing modules and the main processing and communication unit.

The developed hardware setup (Figure 3) includes two MAX30102 sensors [19] connected to a Espressif Systems ESP32-WROOM-32 microcontroller unit (Espressif Systems, Shanghai, China) [20] through a custom-made printed circuit board (PCB). The PCB incorporates the TCA9548A switch (Texas Instruments, Dallas, TX, USA) [21], allowing the device to communicate with multiple Inter-Integrated Circuit (I2C) devices with the same address (in this case, the two sensors). The setup was connected to a Windows computer via a USB port, with a custom-developed software in Python for system control, data acquisition, and processing.

### 3.2. Signal Processing and Analysis

During the acquisition of the PPG signal with the MAX30102 optical sensors, signal processing and analysis algorithms were applied to extract the necessary features from the signal to estimate the vital signs in real time.

The PPG signals obtained with the IR LED of the optical sensors were used for HR and RR estimations due to their lower susceptibility to external factors, making them less noisy compared to the PPG signals obtained with the red LED [22]. One of the factors that affect the PPG signal is the skin tone. However, as reported in [23,24], the skin’s melanin concentration only affects the PPG amplitude, being more significant for the red light, which is more absorbed by this molecule. For the IR light, a decrease in the AC and DC components is also reported, but not as significant. Thus, for the HR and RR measurements, using an IR LED is more suitable than other light wavelengths.

The following subsections provide a detailed description of the signal processing and analysis algorithms applied to the PPG signals for estimating HR and RR values.

#### 3.2.1. Heart Rate Estimation

The frequency of the AC of the PPG signal changes with the HR. Therefore, this vital sign can be estimated by simply identifying the peaks that are related to the heartbeats and the corresponding time in the PPG signal [16].

**Figure 4:**
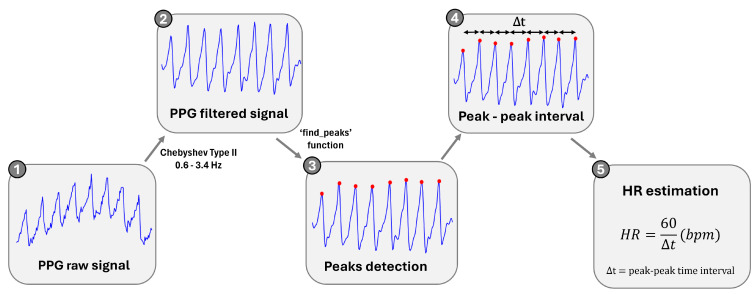
Signal processing block diagram for HR calculation.

To accomplish this, the raw PPG signal was filtered with a 4th-order Chebyshev Type II bandpass filter with cutoff frequencies of 0.6 and 3.4 Hz (corresponding to 36–204 bpm), to minimize the impact of the high-frequency noise on the signal analysis and to eliminate the DC. After filtering, the points of interest (peaks) were identified using the SciPy function ‘find_peaks’ [25]. Knowing the locations of the peaks, the average of the peak-to-peak interval (Δt) was calculated to estimate the HR value. For a better understanding, all the steps of the process are illustrated in Figure 4.

#### 3.2.2. Respiratory Rate Estimation

**Figure 5:**
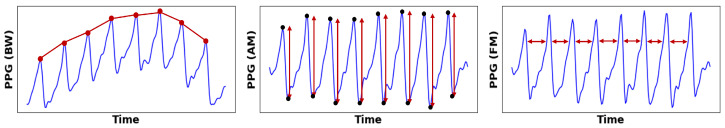
Respiratory modulations that affect PPG signals: BW, AM and FM.

The PPG signal is significantly affected by the respiratory cycle, being modulated in three different ways, as shown in Figure 5 [26,27]:

- Baseline Wander (BW), which corresponds to the shift in the baseline;
- Amplitude Modulation (AM), related with the variations in pulse amplitude;
- Frequency Modulation (FM), related with the variation in the peak-to-peak interval.

Thus, the RR value can be estimated by extracting these signal modulations.

To estimate the RR, the raw PPG signal was filtered, essentially to remove the higher- and lower-frequency components of the PPG. This was achieved by applying a 4th-order Chebyshev Type II bandpass filter (cutoff frequencies 0.067–5 Hz). Next, to extract the three respiratory signals from the filtered signal, the peaks and valleys of the PPG signal, features influenced by respiration, were located using the SciPy function ‘find_peaks’ [25]. The BW, AM and FM signals were then extracted.

**Figure 6:**
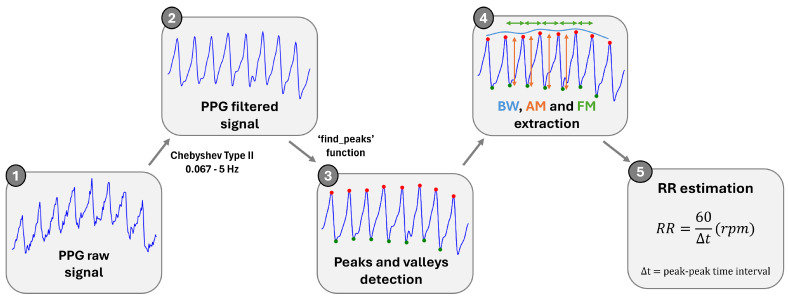
Signal processing block diagram for RR calculation.

The resulting signals were then resampled and filtered, to eliminate the non-respiratory frequencies, using a 4th-order Chebyshev Type II bandpass filter with cutoff frequencies of 0.1 and 0.5 Hz, corresponding to 6–30 rpm. The RR for each of these signals was estimated. First, the peaks of the signals were identified and then the RR was calculated using the average peak-to-peak interval. The RR final value results from the average of the RR values obtained from the three respiratory signals. This process is represented in the diagram shown in Figure 6.

### 3.3. Sensor Position

**Figure 7:**
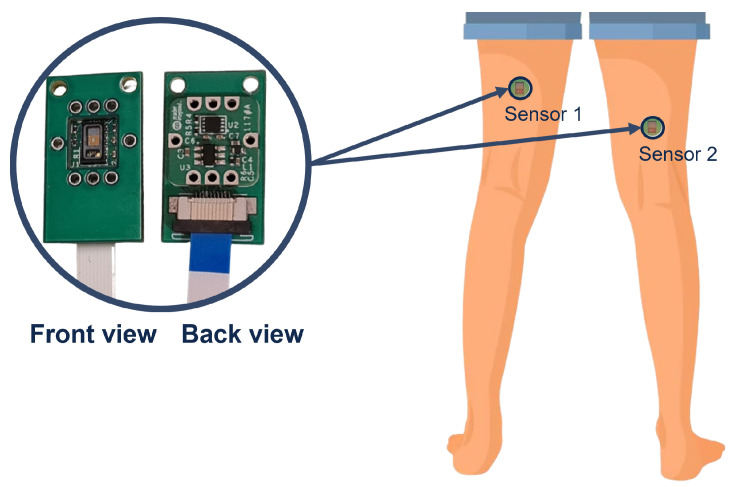
Front and back sides of MAX30102 sensor used in this work and the measurement locations on the back of the thigh.

The back of the thigh is well vascularized, providing good blood flow for reliable PPG signals. However, it differs anatomically and structurally from more commonly used PPG sites, like the fingers. The larger volume of the lower limbs and higher body fat in some areas can weaken the signal and pose challenges in measurement. Thus, these characteristics require additional considerations, such as potential variability in signal quality based on sensor placement. Therefore, to enhance the quality of the acquired signal, a lower region of the back of the thigh, close to the knee joint, was selected, since it has less adipose tissue and the femoral artery and vein and their correspondent branches are more superficial. Additionally, measurements were taken in two locations of this region (Figure 7) to provide measurement redundancy and accommodate individual physiognomic variations.

A transparent polycarbonate interface of 5–6 mm thickness was used between the user’s thigh and the sensor. The optical transmission of this material was studied for the visible and near IR optical windows using a spectrometer and it was possible to observe that this material demonstrated, for red and IR wavelengths, a transmittance close to 90%, which meets the requirement for the developed system and is aligned with our interests.

### 3.4. Volunteer Data Acquisition Protocol

Performance tests were conducted in a total of 17 healthy volunteers (5 males, 12 females) aged between 22 and 40 years old. The volunteers were recruited based on the criteria of being over 18 years of age and having no motor impairments that would prevent them from using the measurement systems.

**Figure 8:**
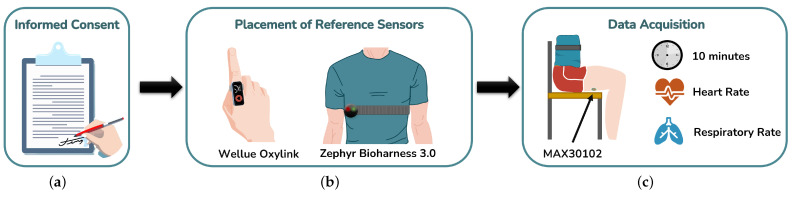
Data acquisition protocol schematic: (**a**) signature of the informed consent, (**b**) placement of the reference sensors in the volunteers’ body and (**c**) data acquisition with the proposed system.

Before taking part in the study, all volunteers were briefed on the entire experimental protocol, and informed consent was signed (Figure 8a).

Volunteers were equipped with two commercial reference sensors—a respiratory band (Zephyr Bioharness 3.0, Medtronic, Lafayette, CO, USA) [28], which was positioned above the chest of the subject, and a PPG device (Wellue Oxylink, Hoofddorp, The Netherlands) [29] attached to one of the subjects’ fingers (Figure 8b). The values collected by these commercial systems were used as the reference values to compare with those obtained from the proposed system.

Each subject was seated in the chair equipped with the optical sensors and instructed to place their legs over the sensors while remaining still and breathing normally. Data were collected in real time for a duration of 10 min, including both the raw and filtered PPG signals, as well as the estimated HR and RR values (Figure 8c).

All data acquisition was performed according to the principles of the Declaration of Helsinki, and the experimental protocol was approved by the Ethics and Deontology Committee of the University of Aveiro, Portugal (No. 49-CED/2023).

## 4. Results and Discussion

**Figure 9:**
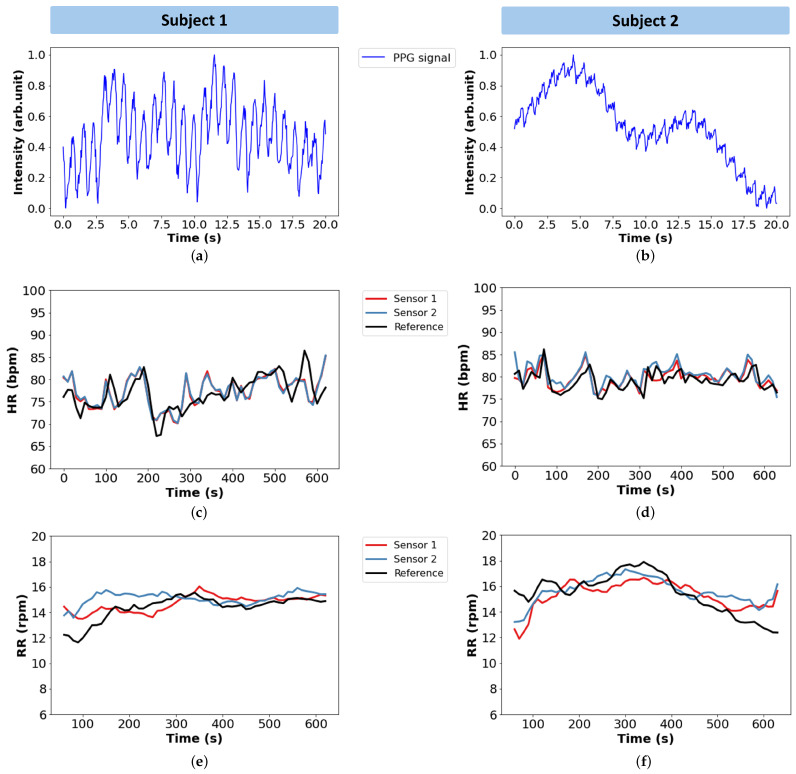
Collected data from two individuals: Subject 1 and Subject 2. The graphs (**a**,**b**) display segments of the PPG raw signal, (**c**,**d**) represent the HR measurements collected during the tests by the two optical sensors and the reference sensor; the same goes for (**e**,**f**) for the acquired RR values.

Figure 9 provides an example of the extracted data from two individuals, referred as Subject 1 and Subject 2. For each subject, three different plots are presented: a 20-s segment of the PPG raw signal, along with graphs displaying the HR and RR measurements collected during testing with both; the developed system and the reference devices.

In Figure 9a,b, the characteristic waveform pattern of the PPG signal is clearly observable, with well-defined peaks and valleys that are crucial for accurate HR and RR extraction. These results confirm that the proposed system is capable of obtaining a PPG signal from the back of the thigh.

HR and RR values over the recording period are represented, respectively, in Figure 9c, Figure 9d and Figure 9e, Figure 9f. Both HR and RR values obtained from the developed system are compared with the data provided by the reference sensors. As observed, for both vital signs, there is a good alignment between the datasets, where the values from the three sensors (two from the developed system and the reference device) follow the same trend over time, supporting the proposed system’s capability to monitor HR and RR under these conditions.

### 4.1. Performance Analysis

Before the performance analysis, the SNR of the PPG signals obtained for each volunteer was evaluated. Low-SNR (<15 dB) [30] data were discarded, aiming to ensure the accuracy and robustness of the study’s conclusions. Through this process, data from volunteers ID9 and ID4 were excluded from the statistical analysis due to the low SNR of the raw PPG signals.

To measure the system’s accuracy for HR and RR monitoring, the Mean Absolute Percentage Error (MAPE) was used. Although no standardized thresholds exist for high or low MAPE, a MAPE > 10 % is usually used in several studies as an indicator of inaccuracy [31,32,33]. The accuracy corresponds to the complement of MAPE, i.e., 100%-MAPE. For MAPE calculation, the ‘mean_absolute_percentage_error’ function from the scikit-learn library was used [34].

#### 4.1.1. Heart Rate Analysis

**Figure 10:**
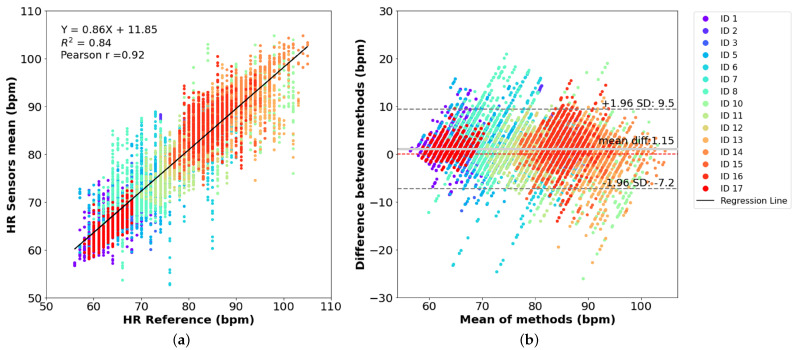
Evaluation of HR measurements: (**a**) correlation plot of the estimated HR and reference HR, (**b**) Bland–Altman plot of the HR data collected by the two systems.

Figure 10a shows the dispersion plot that compares the HR values obtained from the developed system with those measured by the reference. As observed, a strong correlation between the two datasets of readings was obtained, with a Pearson correlation coefficient of r = 0.92 and a determination coefficient of $R^{2}$ = 0.84, indicating an aligned trend pattern between the two systems. The correspondent Bland–Altman plot of HR is shown in Figure 10b. In this diagram, the mean difference between the two groups of data, also called the mean bias, was 1.15 bpm, with limits of agreement between-7.2 bpm and 9.5 bpm. It can be seen that the majority of the points are within the range of ±1.96 standard deviation (SD) and that the mean bias is close to zero, indicating a high agreement between the two systems with no significant bias presented.

For HR measurements, a low MAPE of 4.07 % was achieved, corresponding to a high system accuracy of 95.9 % (>90 %).

#### 4.1.2. Respiratory Rate Analysis

**Figure 11:**
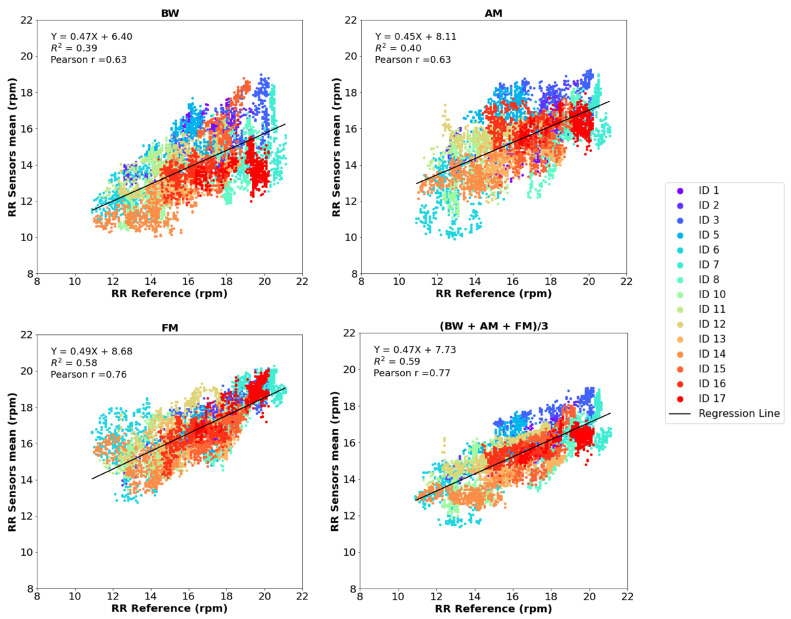
Evaluation of RR measurements: correlation plots of the estimated and reference RR. In the first three graphs, the estimated RR corresponds to the RR obtained for each respiratory modulation (BW, AM and FM), and in the last graph, the estimated RR corresponds to the average of the RR values.

**Figure 12:**
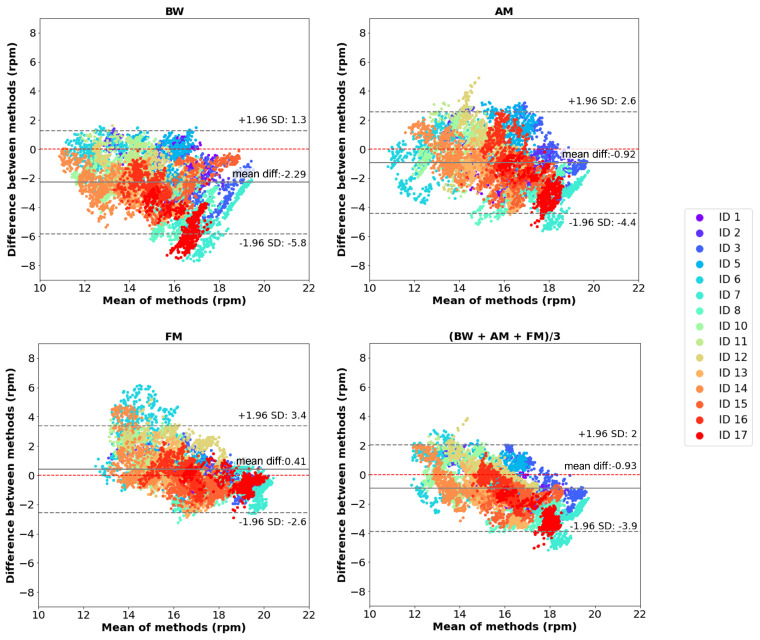
Evaluation of RR measurements: Bland–Altman plots of the RR data collected by the two systems. The first three graphs represent the Bland–Altman plot for the RR estimated with each modulation (BW, AM and FM) and the last graph represents the Bland–Altman plot for the average of the RR values.

The correlation and Bland–Altman plots for RR are presented in Figure 11 and Figure 12, respectively. The three first graphs show the values calculated through each respiratory modulation method: BW, AM and FM, while the last graph displays the RR values representing the average of the three modulation methods.

As illustrated in Figure 11, FM produced the best RR estimates, achieving a Pearson correlation coefficient of r = 0.76 and a coefficient of determination of $R^{2}$ = 0.58. In comparison, the BW and AM methods both yielded a Pearson correlation coefficient of r = 0.63 and coefficients of determination of $R^{2}$ = 0.39 and $R^{2}$ = 0.40, respectively. This difference can be attributed to the fact that BW and AM rely on the amplitude of the PPG signal, making RR estimation more sensitive to movement artifacts present in the PPG data. Overall, the plot showing the average values indicates a good correlation between the two sets of readings, as evidenced by a Pearson correlation coefficient of r = 0.77 and a coefficient of determination of $R^{2}$ = 0.59.

In the Bland–Altman plots for FM, a lower mean difference (0.41 rpm) was obtained compared to the BW (−2.29 rpm) and AM (−0.92 rpm), suggesting a higher agreement between our system and the reference. However, the average plot shows a systematic bias, where the sensor tends to overestimate at RR values below 15 rpm and underestimate the higher ones. This pattern suggests that the sensor’s accuracy varies with the magnitude of RR. However, in general, almost all data are within the upper and lower limits, suggesting a good detection accuracy of the developed system.

These results indicate a lower correlation between the system’s RR measurements and the reference values. This lower accuracy of RR estimation was expected, since RR is not directly extracted from the PPG signal. In contrast to HR, where the PPG waveform is directly linked, making HR calculations more straightforward, RR relies on the respiratory modulations within the PPG signal. Those modulations are less pronounced and happen in lower frequencies than the features used to calculate HR, requiring more complex signal processing algorithms for accurate RR estimation. As a result, RR estimation is highly dependent on the quality of the PPG signal and the algorithms employed, making it more sensitive to noise and motion artifacts present in the signal.

Additionally, as the distance from the heart increases, the physiological mechanisms that cause these respiratory modulations become weaker. This results in less pronounced respiratory modulations in PPG signals collected from the lower limbs compared to those obtained from the upper limbs. Consequently, measuring RR from the legs can affect the accuracy of the calculations [35]. Also, individual factors such as age and respiratory depth can significantly impact the accuracy of RR estimation.

Despite this, for RR measurements, a low MAPE of 8.7% was obtained, corresponding to a high system accuracy of 91.3%.

Nonetheless, it is important to emphasize that the system serves as a proof of concept and that the main objective was to demonstrate that the developed system could successfully acquire PPG signals from the legs and monitor vital parameters like HR and RR using simple signal analysis and processing algorithms. This goal has been achieved, as shown by the obtained results.

## 5. Conclusions

During the present work, a system composed of two optical sensors was successfully developed, based on a MAX30102, to monitor vital signs from the lower limbs in a non-invasive way.

Tests were conducted to assess the capability of the developed system to monitor heart rate and respiratory rate from the lower limbs, and to compare its accuracy with reference devices. Through this tests, it was demonstrated that a photoplethysmography signal can be obtained from the back of the thigh using the proposed system. Although complex signal processing algorithms are required for better estimation, the overall performance of the developed system showed a good Pearson correlation ($r_{HR}$ = 0.92 and $r_{RR}$ = 0.77) with the reference sensor and a high accuracy of the system for HR (95.9%) and RR (91.3%) measurements.

Results confirmed the system’s viability and ability to monitor vital signs such as HR and RR, from the back of the thigh. These findings are important and significant, taking into consideration that the acquisitions were carried out at the back of the thighs, a non-obvious location to measure vital signs. During the tests, movement artifacts were one of the sources of noise in PPG signals and the main challenge in this work.

## Data Availability

The original contributions presented in this study are included in the article. Further inquiries can be directed to the corresponding author(s).

## Acronyms

The following acronyms are used in this manuscript:

AC	Alternating Component
AM	Amplitude Modulation
BW	Baseline Wander
DC	Direct Component
FM	Frequency Modulation
HR	Heart Rate
IR	Infrared
LED	Light-Emitting Diode
MAPE	Mean Absolute Percentage Error
PD	Photodetector
PPG	Photoplethysmography
PCB	Printed Circuit Board
RR	Respiratory Rate
SNR	Signal-to-Noise Ratio
